# Maternal haplotype diversity and phylogenetic relationships of Damascus, Kilis, Hatay, and Kil goat breeds: insight from mtDNA D-loop sequences in the Fertile Crescent

**DOI:** 10.5194/aab-68-607-2025

**Published:** 2025-10-22

**Authors:** İsmail Karaköse, Zühal Gündüz

**Affiliations:** 1 Graduate School of Natural and Applied Sciences, Aydin Adnan Menderes University, Aydin, 09100, Türkiye; 2 Faculty of Agricultural Sciences, Department of Agricultural Biotechnology, Aydin Adnan Menderes University, Aydin, 09070, Türkiye

## Abstract

This study focuses on goat populations in Türkiye, a region located within the Fertile Crescent – historically recognized as the birthplace of goat domestication and a crucial source of genetic diversity in livestock species. This study investigates the maternal genetic origins of four prominent goat breeds – Damascus (DMS, n=20 ), Kilis (KLS, n=21 ), Hatay (HTY, n=22 ), and Kil (KIL, n=21 ) – by analysing 598 base pairs (bp's) of hypervariable region 1 (HV1) within the mitochondrial DNA (mtDNA) displacement loop (D-loop) sequence. The goats utilized in this research were sourced from the “National Breeding Project under Farm Conditions”, a strategic initiative led by the Republic of Türkiye's Ministry of Agriculture and Forestry, General Directorate of Agricultural Research and Policies (TAGEM). The analysis revealed haplotype diversity (Hd) ranging from 0.944 to 0.989 and nucleotide diversity ( π ) between 0.01734 and 0.02635. Hatay goats exhibited relatively low haplotype diversity (Hd = 0.944±0.028 ) and nucleotide diversity ( π=0.01985±0.00087 ). Moreover, the presence of shared haplotypes among multiple individuals in Hatay goats indicates a potential genetic bottleneck or prolonged isolation in breeding compared to other breeds. These findings highlight the necessity for conservation strategies aimed at preserving genetic diversity within this population. Haplogroup A was found to be the most prevalent among the studied populations, a distribution that aligns with the global genetic pattern of maternal origins observed in goat populations worldwide. A total of 51 unique haplotypes were identified, with 47 belonging to Haplogroup A and 4 to Haplogroup G, out of the overall 65 haplotypes. A single shared haplotype was identified between Damascus and Kilis goats. For the first time, Hatay goats have been documented as belonging to both Haplogroup A and Haplogroup G. Additionally, this study presents the novel identification of Haplogroup G in Damascus goats. Furthermore, the phylogenetic analysis also supports the emergence of Kilis and Hatay goats. These findings enhance our understanding of caprine genetic diversity and emphasize the necessity of conserving these breeds as crucial genetic resources within the Fertile Crescent region.

## 1 Introduction

Goat farming occupies a pivotal position within the field of animal production due to its significant contributions to agricultural systems and the multifaceted benefits it provides. Goats are highly adaptable livestock, capable of thriving in diverse and often harsh environments, making them indispensable for food security, rural economies, and sustainable land use. They offer a wide range of products, including milk, meat, fibre, and hides, while also playing a crucial role in ecological conservation and the maintenance of agro-pastoral systems. Scientific investigations have established that goats were among the earliest domesticated animals, with domestication occurring approximately 11 000 years ago in the Fertile Crescent region (Pariset et al., 2011; Naderi et al., 2008; Zeder, 2008; Luikart et al., 2001; Zeder and Hesse, 2000). This region, which encompasses parts of present-day Türkiye, Syria, Iraq, and Iran, is recognized as the birthplace of agriculture and animal domestication (Zeder, 2008; Zeder and Hesse, 2000). Furthermore, the accumulation of mutations over millennia, driven by both natural and human-mediated selection pressures, has led to the emergence of advantageous traits such as disease resistance, heat tolerance, and enhanced production characteristics (Taberlet et al., 2008). The study of these mutations provides valuable insights into the genetic basis of adaptation and the evolutionary history of domesticated animals. The Fertile Crescent is of paramount importance in the context of livestock genetic diversity and the accumulation of mutations, owing to its historical role as the epicentre of animal domestication. The region's diverse environments, ranging from arid deserts to fertile valleys and mountainous terrains, have given rise to numerous locally adapted livestock breeds (Bruford et al., 2003).

Mitochondrial DNA (mtDNA) has become a cornerstone in the study of genetic diversity and evolutionary biology, providing unparalleled insights into the phylogenetic and phylogeographic relationships among species (Naderi et al., 2007; Bruford et al., 2003). Its utility in this research is largely attributed to its distinctive features: strict maternal inheritance, which allows for the tracing of direct maternal lineages; high mutation rates, which provide a rich source of genetic variation for analysis; the absence of recombination, ensuring a clear and unaltered genetic signal; and high copy numbers per cell, which facilitate easier analysis (Pereira et al., 2009; Naderi et al., 2007; Bruford et al., 2003; Luikart et al., 2001). Furthermore, the relatively predictable, clock-like accumulation of mutations in mtDNA serves as a molecular chronometer, enabling scientists to estimate divergence times and reconstruct evolutionary histories with remarkable precision (Luikart et al., 2001). These properties make mtDNA an indispensable tool for exploring the genetic architecture of populations, deciphering migration patterns, and understanding the evolutionary processes that shape biodiversity. The displacement loop (D-loop), which contains the highly polymorphic hypervariable regions I and II (HV1 and HV2), has proven to be particularly valuable in elucidating the origins and evolutionary history of numerous livestock species (Naderi et al., 2007; Bruford et al., 2003). The identification and classification of mtDNA haplogroups in goats were primarily established by Luikart et al. (2001). Through the analysis of mtDNA in domestic goats (*Capra hircus*), numerous researchers have identified six main haplogroups: A, B, C, D, E, and F (Naderi et al., 2007; Liu et al., 2006; Sardina et al., 2006; Pereira et al., 2005; Amills et al., 2004; Joshi et al., 2004; Sultana et al., 2003; Luikart et al., 2001). These haplogroups provide a foundational framework for understanding the domestication processes, geographic dispersal, and genetic diversity of goats. Moreover, these studies have been pivotal in illuminating the origins and evolution of goats, serving as a cornerstone for subsequent research in the field.

According to data from the Turkish Statistical Institute (TUIK, 2024), Türkiye is home to approximately 10.3 million goats, highlighting the significance of goat farming within the country's livestock sector. The various goat breeds present in the country serve as a vital reservoir of genetic diversity, contributing to sustainable agriculture, supporting rural livelihoods, and preserving unique adaptive traits that are crucial for resilience in the face of changing environmental conditions. The Damascus (Shami) and Kil (Hair) goat breeds have played a pivotal role in shaping the livestock systems of the Middle East and Türkiye. Originating primarily from the Middle East, particularly Syria, the Damascus goat is highly esteemed for its superior milk production, reproductive efficiency, and adaptability to hot and arid climates (Mavrogenis et al., 2006). This breed has been extensively raised in southern Türkiye provinces such as Hatay, Gaziantep, Kilis, Şanlıurfa, and Mardin, where it has significantly contributed to the genetic diversity of local goat populations through crossbreeding. Its ability to thrive in challenging environments, combined with its substantial contribution to dairy production, has established the Damascus goat as a key breed in the region. On the other hand, the indigenous Kil goat in Türkiye is renowned for its remarkable resilience to harsh environmental conditions, including rugged terrains and extreme climates. This breed thrives in extensive and semi-intensive farming systems, grazing on marginal lands that are unsuitable for other agricultural activities. The Kil goat's ability to thrive under low-input conditions has made it an essential component of Türkiye's livestock sector. Additionally, it plays a crucial ecological role by preventing soil erosion and maintaining environmental balance in mountainous and semi-arid regions (Daskiran et al., 2018; Keskin et al., 2017b). The adaptability and utility of the Kil goat highlight its importance in the sustainable management of Türkiye's agricultural and ecological systems. The Kilis goat breed, an indigenous genetic resource of Türkiye, has developed through long-term, non-systematic crossbreeding practices between the Damascus and Kil goat breeds by local farmers (Gül et al., 2020). This breed is primarily raised in the southern regions of Türkiye, particularly along the Syrian border, where it has adapted to the local agro-ecological conditions. Recognized as a dairy-oriented breed, the Kilis goat is characterized by its milk production capabilities and is typically managed under extensive and semi-intensive production systems. The breed represents a successful combination of the high milk yield of the Damascus goat and the resilience and adaptability of the Kil goat, achieved through traditional farmer-led breeding practices. Recent scientific investigations have emphasized the importance of the Kilis goat as a critical genetic resource for sustainable livestock production in Türkiye. Studies have extensively documented its phenotypic traits, milk yield potential, and adaptability to marginal environments, highlighting its contribution to the conservation of agricultural biodiversity and the livelihoods of rural communities (Gündüz and Biçer, 2023; Gül et al., 2024, 2018, 2016; Keskin et al., 2017a, 2016). The Hatay goat, also known as the Yayladağ goat, is predominantly found in the Yayladağ district and has been identified as a hybrid of the Kilis goat, exhibiting distinct genetic characteristics (Gül, 2008; Biçer et al., 2005; Kaya, 1999; Keskin and Biçer, 1997). While there is substantial scientific information regarding the phenotypic traits of the Hatay goat, studies exploring its genetic relationships with other regional goat breeds are still limited. This knowledge gap highlights the need for further research to clarify the genetic connections and evolutionary history of the Hatay goat in relation to other breeds, such as the Kilis and Kil goats.

This study aimed to investigate the haplotype diversity, haplogroup composition, and phylogenetic relationships among the Damascus, Kilis, Hatay, and Hair goat populations in Türkiye by analysing the hypervariable region 1 of the mitochondrial DNA D-loop sequence.

## 2 Material and methods

### 2.1 Sample collection

A total of 84 goat blood samples were collected and analysed in this study, which included Damascus goats, also known as Shami goats (DMS, 20); Kilis goats (KLS, 21), Hatay goats, also known as Yayladağ goats (HTY, 22); and Kil goats, also known as Hair goats (KIL, 21). The animal materials for this study were sourced from flocks located in the Hatay Province (including Damascus, Hatay, and Kil goats) and Kilis Province (Kilis goats) as part of the “National Breeding Project under Farm Conditions” coordinated by the Republic of Türkiye's Ministry of Agriculture and Forestry, General Directorate of Agricultural Research and Policies (TAGEM). For each breed, animals exhibiting typical phenotypic characteristics representative of that breed were selected, with careful consideration given to ensuring that the animals were unrelated. Blood samples were collected from the jugular vein of each goat using vacuum tubes containing the anticoagulant ethylenediaminetetraacetic acid (EDTA) (BD Vacutainer Systems, Plymouth, UK), with approximately 9 mL of blood obtained from each animal. The samples were then transported to the laboratory under cold chain conditions to preserve their integrity and were stored at -20 °C until further analysis. This study was conducted in accordance with the approval of the Aydin Adnan Menderes University Animal Experiments Local Ethics Committee, dated 18 May 2023, and numbered 64583101/2023/79.

### 2.2 PCR amplification and sequencing

In this study, genomic DNA was extracted from white blood cells using the high-salt method described by Montgomery and Sise (1990) and Miller et al. (1988), followed by purification. The hypervariable region I (HV-1) of the mitochondrial DNA (mtDNA) D-loop was amplified and sequenced in 84 goats sampled from Türkiye, which is part of the Fertile Crescent. A 598-base pair (bp) fragment, corresponding to positions 15.653 to 16.250 of the goat mitochondrial genome, was successfully amplified using specific primers: forward (CGTGTATGCAAGTACATTAC) and reverse (CTGATTAGTCATTAGTCCATC) (Naderi et al., 2007; Parma et al., 2003). The primer sequences were obtained from the National Center for Biotechnology Information (NCBI) GenBank database (Accession number AF533441.1) to ensure precise targeting of the desired genomic region (Naderi et al., 2007). The PCR reaction mixture comprised 1X PCR buffer, 1.5 mM MgCl_2_, 200 µM of each deoxynucleotide triphosphate (dNTP), 1 µM of both forward and reverse primers, 1 unit of Taq DNA polymerase (Thermo Scientific, USA), and approximately 100 ng of genomic DNA, resulting in a total volume of 30 µL . The amplification process commenced with an initial denaturation at 95 °C for 5 min, followed by a specific number of cycles tailored to the gene of interest. Each cycle consisted of denaturation at 94 °C for 40 s, annealing at 55 °C for 40 s, and extension at 72 °C for 1 min. A final extension at 72 °C for 5 min ensured complete amplification of the PCR products. The amplification was performed using a Bio-Rad C1000 Touch™ Thermal Cycler. To verify the PCR products, the amplified samples were subjected to electrophoresis on a 1.5 % agarose gel, using a 100 bp DNA ladder (Thermo Scientific, 50 bp O'Gene Ruler, USA) as a molecular size marker. DNA sequencing was conducted under a service contract with a reputable provider (Macrogen Inc., Seoul, Republic of Korea), ensuring reliable and high-quality results. The sequences of distinct haplotypes for each goat breed and population have been deposited in the National Center for Biotechnology Information (NCBI) GenBank, with accession numbers ranging from PV068375 to PV068458.

### 2.3 Sequence alignment and data analysis

Nucleotide sequence editing was performed manually using BioEdit v7.2.6 (Hall, 1999), focusing on the removal of ambiguous base calls and low-quality chromatogram regions. Sequences obtained in this study were aligned alongside reference sequences from six known goat mitochondrial haplogroups and outgroup species using the ClustalW algorithm implemented in MEGA 11 (Tamura et al., 2021). Following alignment, trimming was performed in MEGA 11 to remove poorly aligned and non-overlapping end regions, resulting in a final consensus sequence of 453 bp within a 598 bp fragment. This consensus region was defined based on the overlap among all sequences, including the reference and outgroup sequences, to ensure comparability in subsequent phylogenetic and haplotype network analyses. Mitochondrial DNA diversity parameters, including the number of haplotypes (H), haplotype diversity (Hd), nucleotide diversity ( π ), and the number of polymorphic and parsimony-informative sites were estimated for each population and across all populations using DnaSP v6 (Rozas et al., 2017). Reference sequences from the six known goats mitochondrial haplogroups (GenBank accession numbers: AY155721, EF618134 for Haplogroup A; EF617706 for Haplogroup B1; DQ121578 for Haplogroup B2; EF618413, DQ188892 for Haplogroup C; EF617701, DQ188893 for Haplogroup D; DQ241349 for Haplogroup F; EF618535, EF617727, EF618084 for Haplogroup G) were incorporated alongside the sequences obtained in this study. Additionally, sequences from *Capra aegagrus* (GenBank accession numbers: AJ317864, EF989163, EF989426, EF989577), *Capra caucasica* (AJ317875), *Capra sibirica* (AJ317874), *Capra cylindricornis* (AJ317870), *Capra ibex nubiana* (AJ317871), and *Capra falconeri* (AJ317872) were included as outgroups. These sequences were utilized to construct a neighbour-joining (NJ) tree in MEGA 11 (Tamura et al., 2021) using the HKY85+G model (as determined by SMS in PhyML), with 1000 bootstrap replicates, and pairwise deletion for gaps/missing data treatment. A median-joining (MJ) network of mtDNA haplotypes was constructed using NETWORK 10.2 (Bandelt et al., 1999), with a default epsilon value ( ε=0 ), transitions and transversions equally weighted, and median vectors inferred automatically to illustrate the genealogical relationships among haplotypes. The best-fitting nucleotide substitution model was identified using the Smart Model Selection (SMS) algorithm (Lefort et al., 2017) implemented in PhyML 3.0 (Guindon et al., 2010), based on the small-sample-corrected Akaike information criterion (AICc) (Akaike, 1979). This model estimation was performed exclusively to inform the neighbour-joining (NJ) tree construction in MEGA 11. The resulting phylogenetic tree was visualized using FigTree 1.4.4 (Rambaut, 2018). Mitochondrial DNA neutrality tests – including Tajima's D , Fu's Fs, and Fu and Li's D and F statistics – were performed using DnaSP v6 (Rozas et al., 2017). All tests were conducted on the full 453 bp consensus region, using the complete dataset that combined all sampled populations. Statistical significance was evaluated through 10 000 coalescent simulations under the standard neutral model. The genetic differentiation among populations was quantified using Nei's (1972) distance matrix, which was computed in MEGA 11 (Tamura et al., 2021), and subsequently visualized through phylogenetic network analysis in SplitsTree (Hudson and Bryant, 2006).

## 3 Results and discussion

### 3.1 Mitochondrial DNA sequence variation and genetic diversity

We sequenced and analysed hypervariable region 1 of the mitochondrial DNA D-loop in a total of 84 goats representing four breed populations. The nucleotide frequencies were calculated as follows: 30.93 % adenine (A), 30.28 % thymine (T), 22.26 % cytosine (C), and 16.52 % guanine (G). The overall transition / transversion bias ( R ) was estimated to be approximately 76.48, which is higher than the value reported for goat breeds raised in Türkiye ( R=63.74 ) by Kul (2010). Comparative studies have documented transition / transversion ratios R=40 (Wu et al., 2012) and R=36.47 (Chen et al., 2005) for indigenous Chinese goats, R=25 for Vietnam goats, R=28 for Tanzanian goats (Nguluma et al., 2021), and R=17 for indigenous Indian goats (Joshi et al., 2004). The value obtained in this study is higher than values reported for goat breeds raised in Asia. The elevated transition / transversion ratio observed can be attributed to the breeding region of the analysed goat breeds being part of the Fertile Crescent. This finding is particularly important as it highlights the accumulation of mutations within these populations. The bias ( R ) value obtained in this study reflects the mitochondrial DNA D-loop region's tendency to maintain a high transition rate, independent of natural selection, thereby underscoring its evolutionary significance.

As indicated in Table 1, haplotype diversity (Hd) was observed across all breeds, ranging from 0.944±0.028 (HTY) to 0.989±0.019 (DMS). The overall mean haplotype diversity was 0.993±0.003 , indicating a significant level of genetic variation within the populations. In terms of haplotype diversity, the average values obtained in this study were comparable to those reported by Kul (2010) for domestic goat breeds in Türkiye (Hd = 0.998±0.0006 ) and by Akis et al. (2014). However, our values are higher than those reported by Kiraz (2009) (Hd = 0.706±0.019 ) but lower than the data reported by Çakmak (2019) and Kiraz et al. (2024), who found Hd = 1.000. The results obtained in this study are comparable to those reported by Sardina et al. (2006) for the native Sicilian goat breed (Hd = 0.969±0.007 ). Furthermore, our findings align with those of several other studies, including Zhao et al. (2014) for Chinese native goat breeds (Hd = 0.980±0.018 ), Hoda et al. (2014) for Albanian goats (Hd = 0.996), Deng et al. (2017) for Tibetan goats (Hd = 0.990±0.003 ), Diwedi et al. (2020) for Indian native goats (Hd = 0.998±0.001 ), and Nguluma et al. (2021) for Tanzanian goats (Hd = 0.9945±0.001 ). These similarities indicate a relatively high level of haplotype diversity across different goat populations, suggesting substantial genetic variation within these breeds.

Nucleotide diversity ( π ) values range from 0.01734±0.00109 in KIL to 0.02635±0.00336 in DMS, with an average of 0.02091±0.00142 , as shown in Table 1. These moderate nucleotide diversity levels indicate that the populations are genetically diverse; however, there may be some degree of shared ancestry or recent divergence. The relatively higher nucleotide diversity observed in DMS aligns with its greater haplotype diversity, further reinforcing the unique genetic structure of this population. When evaluating nucleotide diversity, the results obtained in this study ( π=0.02091±0.00142 ) align closely with those reported by Kul (2010) for native goat breeds in Türkiye ( π=0.02100±0.00073 ). However, our findings are higher than those of Kiraz (2009) ( π=0.00226±0.00011 ) and Çakmak (2019) ( π=0.01601±0.00006 ). The nucleotide diversity observed in this study is lower compared to the values reported by Kiraz et al. (2024) for Mahalli goats ( π=0.0375±0.00209 ), Nguyen et al. (2022) for Chinese native goats ( π=0.03005±0.00000008 ), Diwedi et al. (2020) for Indian native goats ( π=0.028±0.001 ), Hoda et al. (2014) for Albanian goats ( π=0.036 ), and Zhao et al. (2014) for China local goat breeds ( π=0.0336±0.0008 ). Conversely, our results demonstrate higher nucleotide diversity than those reported for various populations, including Baenyi-Simon et al. (2022) for Congolese goats ( π=0.015±0.003 ), Nguluma et al. (2021) for Tanzanian goats ( π=0.0139±0.046 ), Deniskova et al. (2020) for Russian goats ( π=0.01478±0.00297 ), Deng et al. (2017) for Tibetan goats ( π=0.0145±0.0013 ), and Kamalakkannan et al. (2018) for Indian native goats ( π=0.0088±0.0048 to π=0.0170±0.0088 ). These comparisons indicate varying levels of nucleotide diversity across different goat populations, reflecting differences in genetic variability and evolutionary history.

**Table 1:** Population genetic parameters of the HV-1 region of mtDNA D-loop.

Breed	Sample	Number of	Haplotype diversity	Polymorphic	Parsimony informative	Nucleotide diversity
	size	haplotypes	(Hd ± SD)	sites	sites	( π ± SD)
DMS	20	18	0.989±0.019	54	35	0.02635±0.00336
KLS	21	18	0.981±0.023	53	24	0.02071±0.00350
HTY	22	13	0.944±0.028	38	29	0.01985±0.00087
KIL	21	17	0.981±0.020	42	20	0.01734±0.00109
Mean	84	65	0.993±0.003	95	64	0.02091±0.00142

SD: standard deviation.

The analysis of the aligned combined dataset revealed polymorphism, with 95 polymorphic sites, including 64 parsimony-informative positions. Among the breeds, the DMS goat exhibited the highest number of polymorphic regions (54), while the HTY breed showed the lowest (38). In terms of parsimony-informative sites, the DMS breed also had the highest count (35), whereas the KIL breed had the lowest (20). These findings align with those of Diwedi et al. (2020) but are lower than those reported by Kul (2010) and higher than those observed by Çakmak (2019). The higher number of both polymorphic and parsimony-informative sites in the DMS breed suggests a greater level of genetic variation, which may indicate unique evolutionary characteristics within this population compared to the others.

A total of 65 haplotypes were identified across four goat breeds, consisting of 84 individuals, with 51 of these haplotypes being unique. In haplogroup A, 47 animals exhibited a unique haplotype composition, while haplogroup G contained only 4 animals with unique haplotypes. The proportion of unique haplotypes was highest in the KLS breed (88.9 %), followed by DMS (83.3 %), KIL (76.5 %), and HTY (53.8 %). Upon examining the analysis results, it is evident that two or more animals share the same haplotype, as shown in Table 2. The sharing of haplotypes among multiple individuals within the same breed of HTY goats explains the relatively lower haplotype diversity observed in this breed compared to others. Additionally, it was observed that Haplotype 13 (Hap_13) is shared between individuals from different breeds, specifically between DMS_16, KLS_7, KLS_15, and KLS_16.

**Table 2:** Shared haplotypes among individuals within the same breed.

Haplotype-sharing	DMS	KLS	HTY	KIL
intensity				
2 individuals each	Hap_2	Hap_51	Hap_20	Hap_37
	Hap_12		Hap_24	Hap_42
			Hap_25	Hap_46
			Hap_30	Hap_47
3 individuals each			Hap_26	
4 individuals each			Hap_28	

### 3.2 Phylogenetic analysis

**Figure 1:**
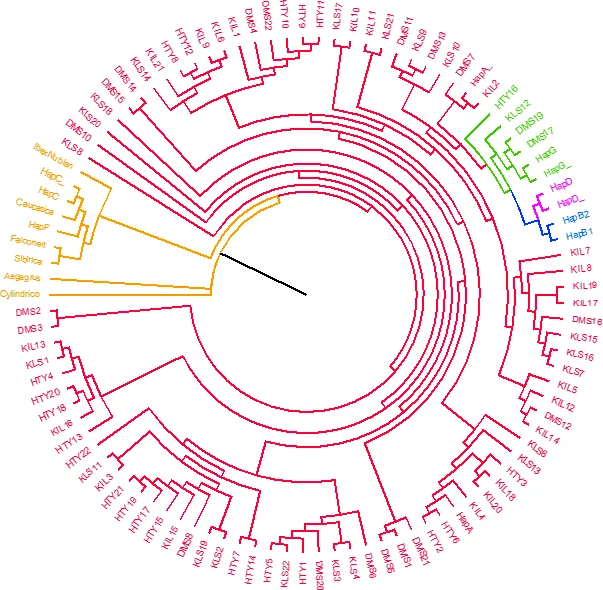
Neighbour-joining tree constructed using HV-1 region of mitochondrial DNA D-loop.

**Figure 2:**
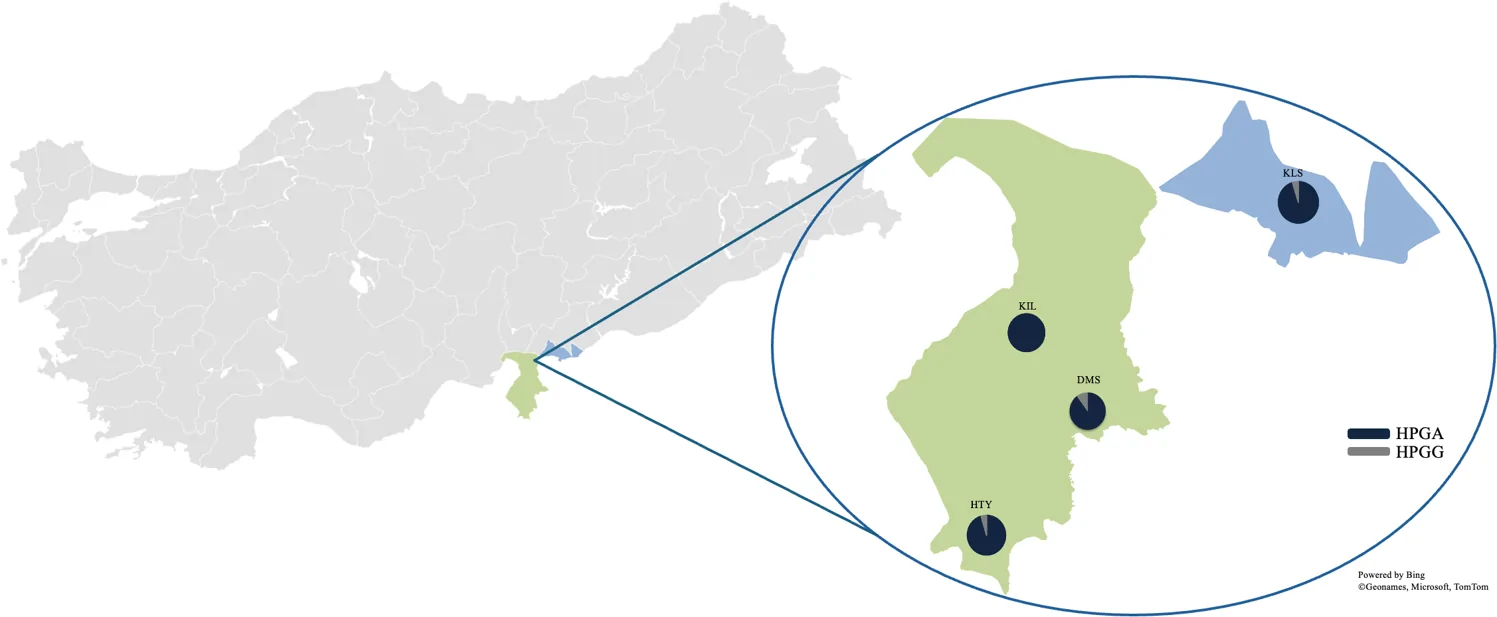
Distribution of goat breeds and mtDNA haplogroups.

In this study, the major mitochondrial DNA haplogroups of goats (A, B, C, D, F, and G) and outgroups were identified and used to construct a neighbour-joining (NJ) tree, which is presented in Fig. 1. The analysis of genetic relationships between the studied goats and reference haplogroups revealed that the breeds are classified into two distinct haplogroups: A and G. The distribution of haplogroups indicated that Haplogroup A comprised the majority, encompassing 80 individuals and 47 haplotypes (95.2 %), while Haplogroup G was represented by only 4 individuals with 4 haplotypes (4.8 %). Figure 2 presents the distribution of haplogroups among breeds and their geographic locations.

The predominance of Haplogroup A observed in this study is consistent with findings reported in the literature (Masila et al., 2024; Bherey et al., 2023; Guo et al., 2022; Baenyi-Simon et al., 2022; Deniskova et al., 2020; Ganbold et al., 2020; Diwedi et al., 2020; Tabata et al., 2019; Ahmed et al., 2016; Zhao et al., 2011; Kul, 2010; Kiraz, 2009; Naderi et al., 2007; Sardina et al., 2006). Similarly, the low frequency of Haplogroup G within the studied populations aligns with previous reports (Deniskova et al., 2020; Kibegwa et al., 2016; Kiraz, 2009; Naderi et al., 2007). Naderi et al. (2007) reported that among 66 goats sampled in Türkiye, 61 belonged to Haplogroup A, while 5 were classified as Haplogroup G. Furthermore, this study was the first to document the presence of Haplogroup G in goat populations from Iran, Saudi Arabia, and Türkiye (specifically among Georgian goats), with an overall frequency of approximately 1 %.

This study represents the first report of Haplogroup G in the DMS goat population in Türkiye, with two individuals classified under this haplogroup, as shown in Table 3. In total, 18 goats from the DMS population were assigned to Haplogroup A. Previous research on DMS goats from Egypt (Othman and Mahfouz, 2016) and Iraq (Al-Abbasi, 2019) has consistently shown that these populations predominantly belong to Haplogroup A. A recent study by Bherey et al. (2023) also identified Haplogroup A as the dominant haplogroup in DMS goats; however, they additionally reported one individual carrying Haplogroup F. Notably, the discovery of Haplogroup G in DMS goats from Türkiye provides new insights into the genetic diversity of this population, highlighting a previously unrecognized aspect of their mitochondrial DNA. In the KLS goat population, 20 individuals were classified under Haplogroup A, while 1 individual was assigned to Haplogroup G. These findings are consistent with previous studies (Kul, 2010; Kiraz, 2009). However, other studies have also identified the presence of Haplogroup D in addition to Haplogroups A and G in Kilis goats (Akis et al., 2014; Kul, 2010). For the first time, this study includes the HV1 mtDNA D-loop region in HTY goats, with 21 individuals classified under Haplogroup A and one individual assigned to Haplogroup G. This marks a significant contribution to the genetic understanding of the HTY goat population. All KIL goats in the present study were assigned to Haplogroup A, which aligns with the findings of previous studies (Çakmak, 2019; Kul, 2010). Also, Kiraz (2009) reported that 15 KIL goats belonged to Haplogroup A, while 1 individual was classified under Haplogroup G.

**Table 3:** Haplogroup distribution of individuals.

Breeds	HPG_A	HPG_G	Number of
			samples
DMS	18	2	20
KLS	20	1	21
HTY	21	1	22
KIL	21	–	21
Total	80	4	84

**Figure 3:**
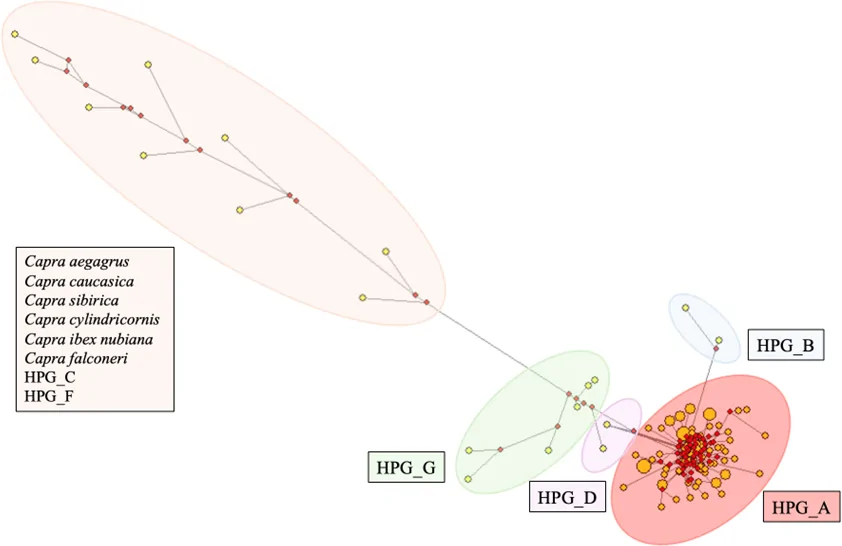
Median-joining network constructed based on the haplotypes.

The median-joining network in constructed by integrating the sequence results of this study with other reference sequences, demonstrating consistency with the previously generated neighbour-joining tree. Figure 3 shows that haplogroups A, B, D, and G are clearly distinguished from each other. The cluster of outgroups (*Capra aegagrus, Capra caucasica, Capra sibirica, Capra cylindricornis, Capra ibex nubiana, Capra falconeri*) and Haplogroup C and Haplogroup F together formed an isolated group on the left side of the median-joining network tree. Large, clustered centres generally represent more common haplogroups, while more distant and isolated clusters represent more rare or specific haplogroups.

### 3.3 Population demographic history

The comparison of DNA sequences within and between populations is a valuable tool for determining the evolutionary factors influencing specific gene regions. It also provides important insights into the species' evolutionary history (Ramos-Onsins and Rozas, 2002). The neutral theory, initially proposed by Motoo Kimura (Kimura, 1968), offers a foundational framework for interpreting DNA variation through well-defined, testable hypotheses. It also provides a suite of statistical tools designed to differentiate the effects of natural selection from those of random genetic drift (Hahn, 2007; Nielsen, 2001; Kreitman, 2000). Tajima's D test (Vitti et al., 2013; Tajima, 1989) is the most widely used general-purpose neutrality test in non-recombinant sequences. Tajima's D is widely regarded as the most effective test in detecting selective sweeps, population bottlenecks, and population subdivision (Simonsen et al., 1995). Tajima's D test is most optimal for detecting an excess of intermediate-frequency alleles and a deficit of low-frequency alleles, whereas Fu and Li's D test is highly sensitive to the presence of very rare alleles (Ferretti et al., 2010). The results of various neutrality tests, including Tajima's D , Fu's Fs, Fu and Li's D , and Fu and Li's F , conducted for the analysed populations are summarized in Table 4. Due to the small number of animals within each haplogroup, neutrality tests were not conducted at the haplogroup level but were instead analysed at the breed level and for the overall population.

**Table 4:** Demographic parameters estimated from HV1 of mtDNA D-loop analysis.

Breeds	Tajima's D	Fu's Fs	Fu and Li's D	Fu and Li's F
DMS	-0.94279	-6.053	-0.39076	-0.64879
KLS	-1.45534	-6.636	-1.52237	-1.75427
HTY	-0.53528	-0.761	0.30062	0.05334
KIL	-1.29220	-6.143	-1.36149	-1.56481
Mean	-1.70157	-59.074	1.50810	-1.90492

Tajima's D values and Fu's Fs estimates for all goat populations were negative and statistically non-significant. The negative values observed in Tajima's D test for all breeds indicate that these populations have not deviated from neutral population values. In general, the higher-than-expected number of rare alleles at low frequencies could suggest a potential population expansion or the influence of negative selection pressure. Similarly, the negative values observed in Fu's Fs test suggest the presence of population expansion or selective pressure. Particularly, the very high negative value ( -59.074 ) for the general population indicates that these processes are highly pronounced across the populations. When examining Fu and Li's D and F statistics in the DMS, KLS, and KIL populations, the negative values indicate the presence of rare alleles and suggest the influence of population expansion or negative selection. However, the positive values of Fu and Li's D and F in the HTY population point to a scarcity of rare alleles and suggest the potential mild effects of a population bottleneck.

### 3.4 Genetic distance among breeds

**Figure 4:**
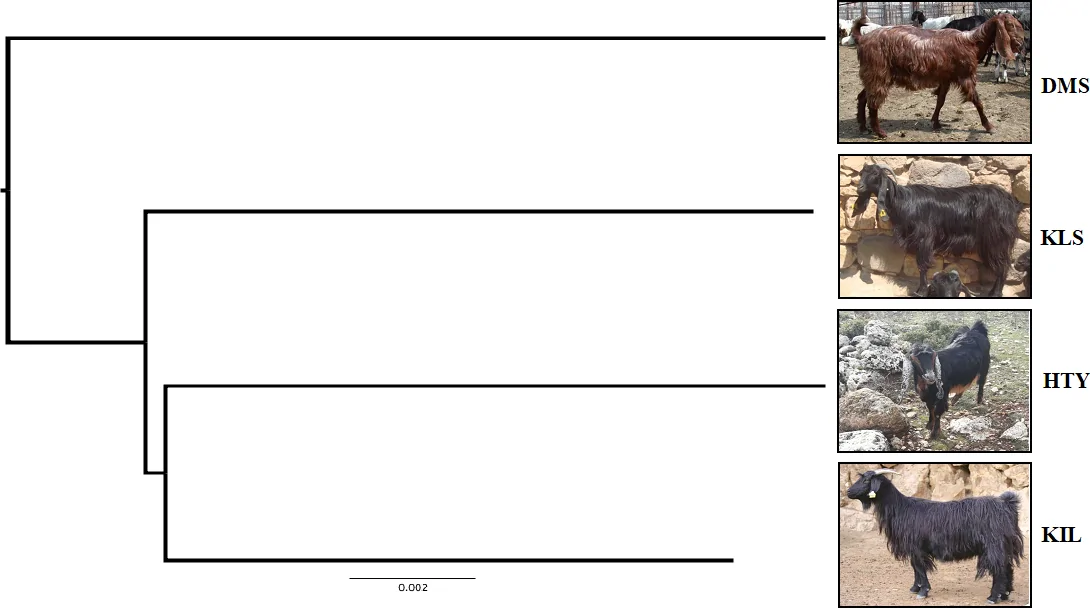
The dendrogram based on Nei's distance matrix for the populations.

The genetic distance between populations was computed using Nei's (1972) distance matrix (Table 5), and the resulting dendrogram is shown in Fig. 4.

The DMS goat population is positioned at the greatest genetic distance from the KLS, HTY, and KIL goat populations. The populations that are closest to each other are HTY and KIL, followed by KLS. Previous studies have reported that the KLS population emerged as a result of uncontrolled crossbreeding between DMS and KIL goats by local breeders aiming to increase milk and meat production (Gündüz and Biçer, 2023; Gül et al., 2020; Keskin, 2002). According to the dendrogram, the KIL and HTY goat populations are closely related. The proximity of the HTY goat genotype, resulting from the crossbreeding of KLS and KIL goats (Gül, 2008; Biçer et al., 2005; Kaya, 1999; Keskin and Biçer, 1997), to the local KIL population is expected.

**Table 5:** Nei's distance matrix for the populations.

	DMS	HTY	KIL	KLS
DMS	–	0.0038	0.0035	0.0037
HTY	0.0260	–	0.0033	0.0034
KIL	0.0244	0.0195	–	0.0031
KLS	0.0257	0.0213	0.0200	–

Da (genetic similarity) is located above the diagonal, and Dxy (genetic distance) is located below the diagonal.

## 4 Conclusion

The findings of this study provide a comprehensive understanding of the genetic diversity and phylogenetic relationships among goat breeds in Türkiye, as revealed by mitochondrial DNA markers. The observed average haplotype diversity value (Hd = 0.993±0.003 ) ranks among the highest reported globally, highlighting the significant genetic variability within these populations. Phylogenetic and network analyses identified the presence of two globally recognized haplogroups, A and G. This study presents the first identification of haplogroups in Hatay goats and reports the presence of Haplogroup G in Damascus goats for the first time. The relatively low haplotype diversity (Hd = 0.944±0.028 ) and nucleotide diversity ( π=0.01985±0.00087 ) observed in Hatay goats suggest that this population has limited genetic diversity compared to other breeds. Additionally, the sharing of identical haplotypes among multiple individuals indicates that this population may have undergone a genetic bottleneck or possesses restricted diversity in maternal lineages. These findings imply that the population may have descended from a small number of ancestors or has been bred in isolation for an extended period. Such insights are valuable for informing conservation programmes and maintaining genetic diversity. The dendrogram constructed from Nei's genetic distance matrix supports the phylogenetic relationships among the studied breeds. These results not only contribute to the broader knowledge of caprine genetic resources but also underscore the importance of conserving these breeds as valuable genetic reservoirs within the Fertile Crescent region.

## Supplement

10.5194/aab-68-607-2025-supplement The supplement related to this article is available online at https://doi.org/10.5194/aab-68-607-2025-supplement.

## Data Availability

This article is derived from İsmail Karaköse's master's thesis conducted under the supervision of Zühal Gündüz at Aydin Adnan Menderes University. The sequences of the Damascus, Kilis, Hatay, and Kil goat breeds generated in this study are available in the NCBI GenBank repository, with accession numbers ranging from PV068375 to PV068458.
