# Supplementary material for: Maternal haplotype diversity and phylogenetic relationships of Damascus, Kilis, Hatay, and Kil goat breeds: insight from mtDNA D-loop sequences in the Fertile Crescent
Source: Arch Anim Breed. 2025 Oct 22;68(4):607–18. doi: 10.5194/aab-68-607-2025 (PMC13494912; doi:10.5194/aab-68-607-2025)
Supplement: Supplementary file 1 [file aab-68-607-2025-supplement.zip › aab-68-607-2025-supplement-title-page.pdf]

Supplement of Arch. Anim. Breed., 68, 607–618, 2025
https://doi.org/10.5194/aab-68-607-2025-supplement
© Author(s) 2025. CC BY 4.0 License.

Open Access
Archives Animal Breeding

Supplement of

Maternal haplotype diversity and phylogenetic relationships of Damascus, Kilis, Hatay, and Kil goat breeds: insight from mtDNA D-loop sequences in the Fertile Crescent

İsmail Karaköse and Zühal Gündüz

Correspondence to: Zühal Gündüz (zuhal.gunduz@adu.edu.tr)

- aab-68-607-2025-supplement-title-page.pdf
- Table S1_Supplementary data.xlsx

The copyright of individual parts of the supplement might differ from the article licence.
